# Femtosecond Laser Direct Writing of Gecko-Inspired Switchable Adhesion Interfaces on a Flexible Substrate

**DOI:** 10.3390/mi14091742

**Published:** 2023-09-06

**Authors:** Zhiang Zhang, Bingze He, Qingqing Han, Ruokun He, Yuxuan Ding, Bing Han, Zhuo-Chen Ma

**Affiliations:** 1 Department of Automation, Shanghai Jiao Tong University, Shanghai 200240, China; 2 Institute of Medical Robotics, School of Biomedical Engineering, Shanghai Jiao Tong University, Shanghai 200240, China

**Keywords:** switchable adhesion, two-photon polymerization, mushroom-shaped micropillars

## Abstract

Biomimetic switchable adhesion interfaces (BSAIs) with dynamic adhesion states have demonstrated significant advantages in micro-manipulation and bio-detection. Among them, gecko-inspired adhesives have garnered considerable attention due to their exceptional adaptability to extreme environments. However, their high adhesion strength poses challenges in achieving flexible control. Herein, we propose an elegant and efficient approach by fabricating three-dimensional mushroom-shaped polydimethylsiloxane (PDMS) micropillars on a flexible PDMS substrate to mimic the bending and stretching of gecko footpads. The fabrication process that employs two-photon polymerization ensures high spatial resolution, resulting in micropillars with exquisite structures and ultra-smooth surfaces, even for tip/stem ratios exceeding 2 (a critical factor for maintaining adhesion strength). Furthermore, these adhesive structures display outstanding resilience, enduring 175% deformation and severe bending without collapse, ascribing to the excellent compatibility of the micropillar’s composition and physical properties with the substrate. Our BSAIs can achieve highly controllable adhesion force and rapid manipulation of liquid droplets through mechanical bending and stretching of the PDMS substrate. By adjusting the spacing between the micropillars, precise control of adhesion strength is achieved. These intriguing properties make them promising candidates for various applications in the fields of microfluidics, micro-assembly, flexible electronics, and beyond.

## 1. Introduction

Switchable adhesion interfaces (SAIs) with dynamic adhesive states and stimulus-responsive properties have gained widespread attention across diverse disciplines in the past decade [1,2]. These adhesives hold significant promise for applications such as pain-free detachment [3,4,5], manipulation of non-planar objects with smart grippers [6,7], precision microscale arrays [8,9], and flexible electronics [10,11,12]. SAIs simplify manipulation processes and have been integrated into wireless soft robots for in situ inspection tasks [13], revealing their potential in clinical practice. To develop SAIs with desired properties, researchers have sought inspiration from various creatures, such as geckos [14,15,16,17], frogs [18], octopuses [19,20], mussels [21,22], remora [23,24], and beetles [25,26], to construct bio-inspired interfaces with dynamic adhesive capabilities.

Among various bio-inspired switchable adhesion interfaces, those inspired by geckos are considered the most promising due to their adaptability to extreme environments [27], insensitivity to small defects [28], and high theoretical bonding strength [29]. Gecko footpads, with hierarchical hair arrays considered the source of their outstanding adhesion capabilities, can reduce effective elastic modulus through nanoscale splitting, enabling conformal contact with surfaces [30,31]. These gecko-inspired interfaces have demonstrated robust adhesion strength exceeding 100 kPa [32,33]. However, their high strength hinders flexible control, lacking a comparable neuro-muscular system found in geckos’ footpads.

Previous efforts to address this issue involve incorporating stimulus-responsive materials like liquid crystal elastomers [34], shape memory polymers [35], MoO_3−x_ [16], graphene [17], low-melting-point alloys [36], and Ni-Ti alloys [37] into the gecko-inspired adhesive to modulate stiffness or deformation. However, phase transition or deformation processes are typically slow, sometimes taking more than 60 s. More importantly, external stimuli such as ultraviolet radiation [38] or high temperatures [5] may cause unpredictable damage to the target surfaces. Despite employing magnetic silicone to fabricate gecko-inspired adhesives that can achieve rapid response [39], shrinking them down to the microscale causes sharp declines in deformability and switching ability, as both magnetic force and torque decrease with the second to the fourth power of size [40,41,42]. Another method to accelerate the detachment process relies on mechanical deformation, such as sliding [43], twisting [44], and buckling [45], of the adhesion microstructures under overload. This method can achieve an ultra-high switching ratio, but the overload force is usually significantly higher than the force of preload, implying that releasing objects on soft substrates or in the air without sufficient support are impossible.

In contrast, more elegant approaches involve stretching and bending the adhesive mechanically, closely resembling the natural gecko’s switching mechanism, where adhesion switching is accompanied by footpad stretching and bending [46,47]. A flexible substrate is crucial for implementing such adhesion mechanisms. Traditional gecko-inspired adhesives obtained through replica molding limit structures to 2.5D, resulting in constrained adhesion strength and applicability [48]. Femtosecond laser direct writing (FsLDW) enables the fabrication of complex three-dimensional structures, yet few soft materials have been explored, and their adhesion performance on flexible substrates remains unexplored [33].

In this study, we employ FsLDW to fabricate three-dimensional mushroom-shaped soft PDMS micropillars on a PDMS flexible substrate. The high spatial resolution allows precise construction of the mushroom with arbitrary head size. Stretching or bending the flexible substrate mechanically accurately modulates the spacing among mushroom-shaped micropillars. Increasing spacing reduces the effective contact area and adhesion force under similar preloads, while reducing spacing yields an inverse effect. Our adhesive exhibits outstanding switchable adhesion capabilities for both solid and liquid targets. The compatibility of these micropillars with the substrate ensures multiple deformation cycles without unexpected detachment due to deformation mismatch. Our work presents a novel strategy for developing adhesion surfaces with rapid, non-destructive, and highly controllable features, with promising implications in micro-assembly, microfluidics, flexible electronics, and beyond.

## 2. Materials and Methods

### 2.1. Materials

IP-PDMS (Nanoscribe GmbH, Eggenstein-Leopoldshafen, Germany) was used for the FsLDW of gecko-inspired microstructures. Indium tin oxide (ITO)-coated soda–lime substrates were also purchased from Nanoscribe GmbH. Silicone elastomer (PDMS, Sylgard 184, Dow, Beijing, China) was used as received without further modification to construct the flexible substrate. Isopropanol (IPA, 99%, Aladdin, Shanghai, China) was employed as the developer solution.

### 2.2. Methods

To fabricate the flexible substrate, the PDMS monomer and the curing agent were mechanically stirred at a ratio of 10:1 for minutes. Subsequently, the PDMS was subjected to vacuuming for 15 min to remove any bubbles. The de-aerated PDMS was then spin coated on the ITO substrate at 500 rpm for 30 s. Afterward, it was transferred to a hot oven and baked at 55 °C overnight.

A commercial FsLDW system (Photonic Professional GT2, Nanoscribe GmbH & Co. KG, Eggenstein-Leopoldshafen, Germany) was employed for exposing IP-PDMS into mushroom-shaped micropillars. First, the SolidWorks 2021 software (Dassault Systemes, Ile-De-France, France) was employed to design the mushroom-shaped micropillars with desirable geometries and generate corresponding STL files. Subsequently, the Describe 2.7 software (Nanoscribe) was utilized to create GWL files from STL files, in which the slicing and hatching distances are both set to 0.3 µm. The GWL files were then executed within Nanoscribe, where the 25× objective (NA = 0.8; WD = 380 µm; Carl Zeiss AG, Oberkochen, Germany) was employed to focus the femtosecond laser on the pre-prepared PDMS substrate with a drop of IP-PDMS resins on it. The FsLDW system is set as a dip-in laser lithography mode (i.e., dipping the objective into the resin), with a scanning speed of 80 mm/s. The center wavelength of the femtosecond laser is 780 nm, with a pulse duration of 80–100 fs, calibrated laser power of 50 mW, and repetition rate of 80 MHz. During the writing, the coordinates in the xy-plane within one print field are addressed using the Galvo scanner, while the position along the *x*-axis is managed by the piezo stage. Based on the aforementioned parameters, the writing time for each mushroom-shaped micropillar is ~15 s. The printing efficiency of microstructures can be freely adjusted further by varying the slicing distance, hatching distance, and scanning speed under different resolution requirements. Upon completion of the writing, the sample was transferred to the ethanol bath for development for 10 min until excess IP-PDMS resin dissolved. After being dried by compressed air, the sample was peeled off from the ITO carefully.

The scanning electron microscopy (SEM) images were captured by NOVA NanoSEM 230 (FEI, Culver City, CA, USA). Surface roughness on the tip of a single micropillar was measured by an atomic force microscope (AFM) (Fastscan Bio, Bruker, Billerica, MA, USA). The optical photographs were taken with an upright microscope (MJ43, Mshot, Guangzhou, China). The contact-angle measurements were performed using the Theta Flex instrument (Biolin Scientific, Gothenburg, Sweden). The adhesion-force testing of BSAI was conducted on a customized platform [49], mainly comprising a load cell (GSO-25, Transducer Techniques, Temecula, CA, USA), a force probe (ACL108U, Thorlabs, Newton, NJ, USA), a signal conditioner (TMO-2, Transducer Techniques, Temecula, CA, USA), a data acquisition board (USB-6001, National Instruments, Austin, TX, USA), and an inverted microscope (MI52-N, Mshot, Guangzhou, China). To minimize viscoelastic effects, both the approach and retraction speeds of the probe were set to 10 µm/s. The stretching and bending of the BSAI were achieved by clamping them onto a micrometer caliper.

## 3. Results

### 3.1. Microfabrication of BSAI on a Flexible Substrate

**Figure 1:**
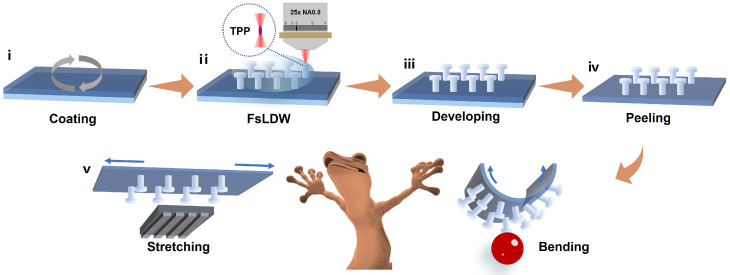
Schematic FsLDW of BSAI on a flexible substrate. (**i**) PDMS spin-coated onto the ITO substrate, (**ii**,**iii**) FsLDW and developing of the mushroom-shaped micropillars based on IP-PDMS, (**iv**) Peeling the micropillar arrays film off the substrate, and (**v**) illustration of sample bending and stretching along with promising applications.

According to third-order nonlinear optical effects, only in regions of sufficiently high optical intensity, such as the focal point of a laser, can the simultaneous absorption of two photons lead to two-photon polymerization (TPP). Based on the high spatial resolution of TPP, FsLDW has exhibited great advantages in the fabrication of intricate microstructures [50]. Despite that, from the perspective of materials, generally, the material modules of microstructures fall within the range of either GPa [51] or kPa [52]. Such a fact results in these microstructures having limited mechanical performances [53]. Employing materials with moderate modulus can achieve a smart equilibrium between high precision and flexibility in microstructure fabrication, which is especially critical for providing excellent adhesion performance. To this end, IP-PDMS, a highly biocompatible and soft negative resin, is utilized as the matrix of microstructures in this paper. Despite the fact that the composition of the IP-PDMS has not been fully divulged owing to commercial issues [54], its formation mechanism is clearly based on TPP during FsLDW. The microstructures based on such resin possess a Young’s modulus of ~15 MPa [55], which is three orders of magnitude lower than conventional acrylic-based resins and matches very well with the PDMS substrate [56]. Such a fact implies that the microstructures and the substrate can form a favorable connection at the interface and enable them to undergo consistent deformations when the substrate is stretched or bent, thereby demonstrating the potential to manipulate both solid and liquid targets, as exhibited in Figure 1. Notably, such mushroom-shaped micropillars with negative slopes were usually difficult to replicate, owing to the increased risk of structural damage during the molding process [57]. Our processing strategy overcomes the spatial limitations of real three-dimensional interfaces while ensuring the robustness of the gecko-inspired adhesives, which further expands the design space of BSAIs.

### 3.2. Geometric Characterizations of BSAIs

**Figure 2:**
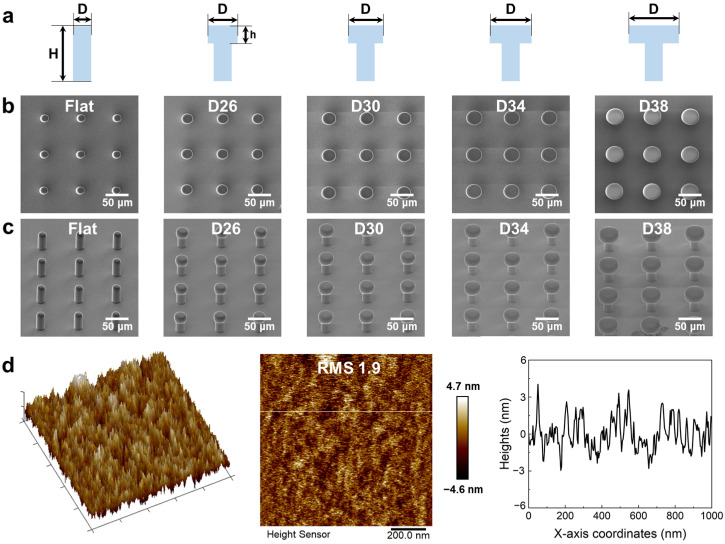
Geometries of micropillars with different tip diameters. (**a**) Design dimensions of the micropillars. (**b**) SEM images of micropillar arrays from top view. (**c**) SEM images of micropillar arrays from side view. (**d**) AFM topographies on the tip of a mushroom-shaped micropillar.

To fabricate microstructures with desired functionalities, a series of mushroom-shaped micropillars with different tip diameters were designed. Consistently, every sample possesses a stem diameter of 18 µm, height of 50 µm, and pitch of 75 µm. The tip diameters of the mushroom-shaped micropillars are varied from 26 to 38 µm, as shown in Figure 2a. After the development process, SEM was employed to characterize the geometric morphologies of the microstructures. As depicted in Figure 2b,c, the tips of mushroom-shaped micropillars had extremely smooth top surfaces. Such a fact was further confirmed by AFM morphology testing (Figure 2d). The AFM results indicate that the average surface roughness on the tip of a mushroom-shaped micropillar is ~1.9 nm, which even surpasses that of some near-perfect diffractive optical elements (~10 nm) [58]. These results demonstrate that FsLDW enables the mushroom-shaped micropillar with the capacity to form good conformal contact with the target surface, which was considered the foundation of excellent adhesion properties. However, too large tip diameters are good for solid adhesion, but not desirable for droplets. To achieve a switchable hydrophobic character, mushroom-shaped micropillars with a tip diameter of 30 µm were used in subsequent studies. This trade-off is reasonable as an excessively large solid contact area would increase the danger of uncontrollable wetting of the sample [59].

### 3.3. Deformation Characterizations of BSAIs

**Figure 3:**
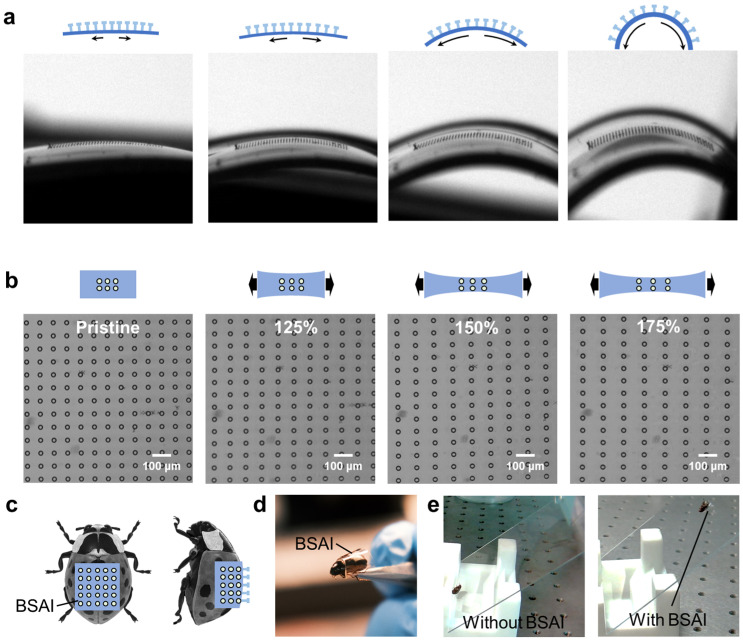
Deformation characterizations of the micropillar arrays on a flexible substrate. (**a**) Schematic and corresponding real experiment snapshots of bending BSAI with increased curvatures. (**b**) Schematic and corresponding real experiment snapshots of bending the sample with increased stretching strains. (**c**) Illustration of a beetle equipped with flexible BSAI. (**d**) A photograph of a beetle specimen equipped with flexible BSAI. (**e**) The state of beetle specimen equipped with (right) and without (left) flexible BSAI on an inclined glass.

To further demonstrate the connection robustness of mushroom-shaped micropillars on flexible substrates, the BSAIs were secured onto a vernier caliper using a clamp. By manually adjusting the distance between the measuring jaws, the morphology of the flexible substrate could be precisely controlled, and thereby the pitch among micropillars on it. In detail, as the measuring jaws gradually close, the substrate can be bent upwards with increasing curvature, thereby increasing the pitch among the mushroom-shaped micropillars on it (Figure 3a). On the other hand, increasing the distance between the measuring jaws resulted in the stretching of the substrates. With the gradual stretching of the sample, the pitch among the micropillars exhibited a responsive increase as exhibited in Figure 3c. Even at a stretching strain of 175%, the micropillars remained adhered to the substrate without signs of collapse. These results indicate the remarkable mechanical robustness of the BASI during stretching and bending [60], which is of great importance in achieving adhesion functions in practical applications, especially in robotics interfaces. Taking the beetle’s dorsal surface as an example, the originally smooth surface was equipped with as-made adhesive, as shown in Figure 3c,d. On the inclined glass, the pristine beetle specimen quickly slips off (Figure 3e, left), whereas the one equipped with the flexible adhesive can adhere steadily (Figure 3e, right). In the future, equipping BSAI with controllable soft robots rather than specimens is envisioned to achieve cross-species functionalities.

### 3.4. Force Characterizations of BSAIs

**Figure 4:**
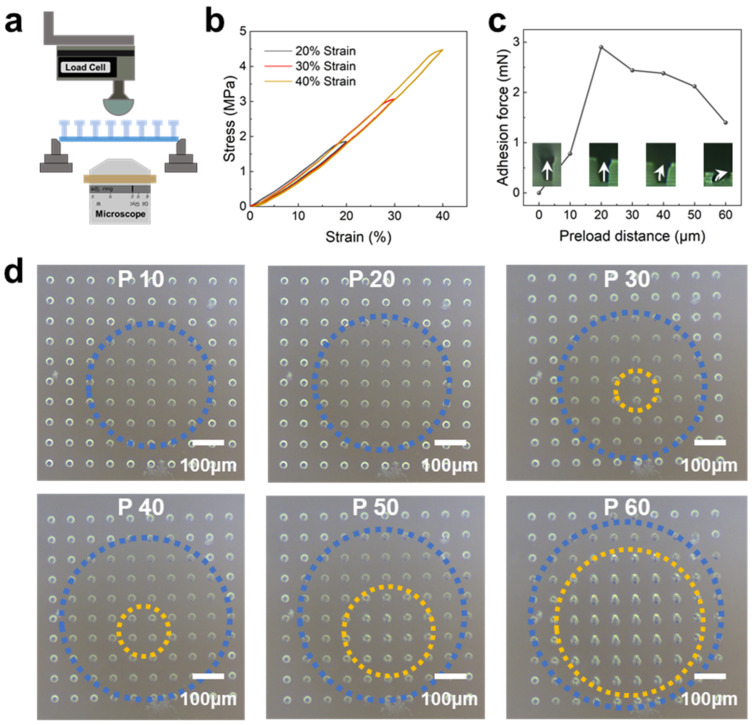
Force characterizations in non-stretched BSAIs. (**a**) Illustration of the custom adhesion platform. (**b**) Stress–strain curves of a single micropillar on the flexible substrate. (**c**) Adhesion values of the non-stretched samples under different preloads and the corresponding morphologies of the micropillars. (**d**) The evolutions of the corresponding contact signatures of the non-stretched samples under different preloads. The regions enclosed by the blue dashed line represent the efficient contact areas, while the regions enclosed by the yellow dashed line indicate the collapse areas of the BSAIs.

As illustrated in Figure 4a, a custom-made testing platform was installed on an inverted microscope to assess the force characterizations of non-stretched BSAI. We first identified the stress–strain behavior of a single micropillar on the flexible substrates. Even at a strain of 40%, we found that the stress–strain curve exhibited no signs of snap-through instability (Figure 4b). This result further indicates the robustness of mushroom-shaped micropillars on flexible substrates. The adhesion versus preload curve is used to decide the optimal preload, as shown in Figure 4c. It should be noted that the measured adhesion refers to the force on the entire contact surface rather than a single micropillar. As the preload increased from 0 to 20 µm gradually, the adhesion force of BSAI also improved from 0 to 2.93 mN. However, further increments in preload cause a decrease in the adhesion force: at a preload of 60 µm, the adhesion force decreased to 1.39 mN. Such magnitude of reduction aligns with previous reports that employ pillars of millimeter scales to construct adhesion interfaces [61]. In situ observation of the deformations of micropillars at different preloads by the inverted microscope further aided in understanding the underlying reasons. During the loading process, the perceptible variations in the color and morphology of mushroom-shaped micropillars could be employed to identify the contact states [45]: non-loaded micropillars exhibit high brightness; slight-loaded micropillars show darkening colors; overloaded micropillars regain brightness due to the reduction in contact area. These variations of morphology under different loads are strongly consistent with the inserted side-view photos in Figure 4c. As exhibited in Figure 4d, the contact area at a preload of 20 µm was significantly larger than that at 10 µm, implying the improved adhesion force. Further increasing the preload caused the local collapse of micropillars and insufficient contact, which is consistent with the reduced adhesive force [62]. It is noteworthy that even under severe overloading (i.e., 60 µm), the micropillars can retain their pristine geometries and regular arrangement after removing the preload completely, further demonstrating the robust connection between the micropillars and the substrate. Despite no damage to the micropillars, an appropriate preload is still necessary to maximize the adhesion force of non-stretched BSAI. Hence, a preload of 20 µm is considered to be an optimal parameter in the following adhesion characterizations. The adhesion forces of BSAIs with different heights under the same preload (20 µm) are also available in Figure S1, which demonstrates that a height of 50 µm is also reasonable for achieving high adhesion force.

**Figure 5:**
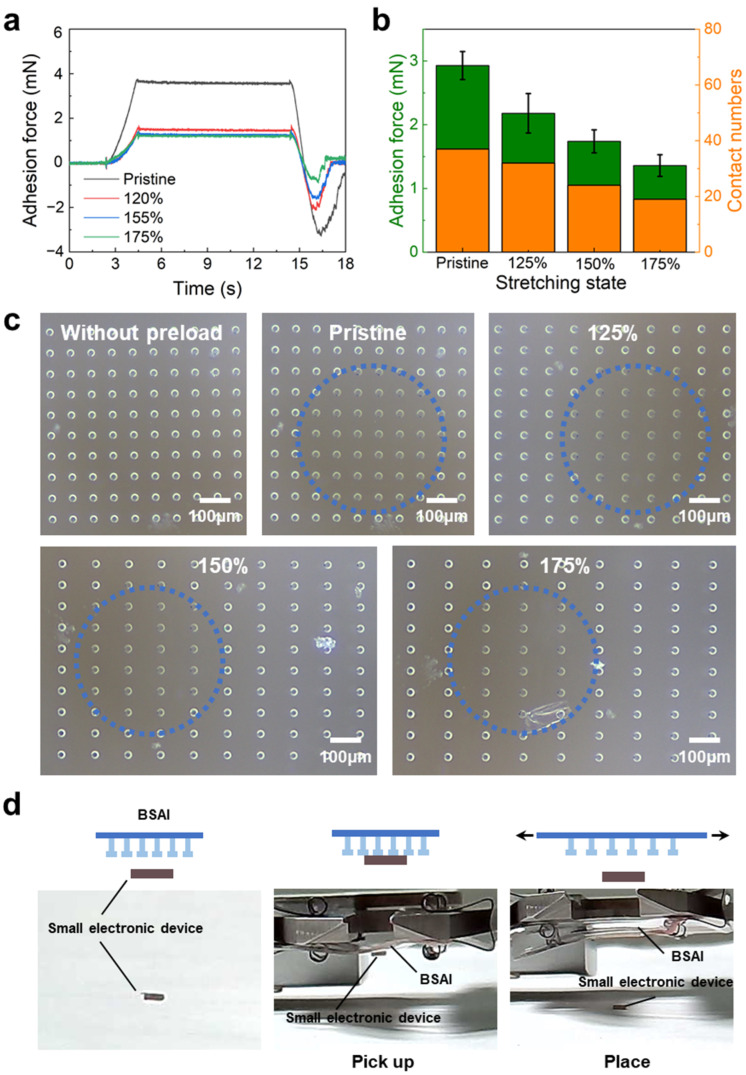
Adhesion characterizations in stretched BSAIs. (**a**) Adhesion force graphs with respect to time of the samples under different stretching strains. (**b**) Adhesion values of the samples under varied stretching strains (**c**) The evolutions of the corresponding contact signatures with corresponding stretching strains. The region enclosed by the blue dashed line represents the efficient contact area. (**d**) The application of BSAI in non-destructive transfer of a small electronic device.

Next, the adhesion forces of BSAIs were measured under different stretching strains, as shown in Figure 5a. At a stretching strain of 175%, the adhesion force was reduced to 1.36 mN (Figure 5b), which is significantly lower than the adhesion force of non-stretched BSAI. The underlying mechanism for this reduction was again revealed by in situ microscope observation, as shown in Figure 5c. As the sample was stretched, the number of micropillars within the region enclosed by the blue dashed line, in which the probe could establish contact, decreased gradually. The number of micropillars in efficient contact was quantified and is presented in Figure 5b, which exactly followed the same trend as the adhesion force. These results demonstrate that stretching BSAI leads to a gradual reduction in the number of micropillars in efficient contact with the target, thus influencing the magnitude of the adhesion force. Based on that, such BSAI enables the non-destructive transfer of fragile and thin objects. For instance, we demonstrated the process of picking up and placing a small electronic device (2.0 × 1.2 × 0.8 mm) by using BSAI, as exhibited in Figure 5d. This characteristic allows it to be potentially applied to non-destructive transfer printing of fragile and thin objects, such as LEDs [11] and other flexible electronics [63].

### 3.5. Wettability Characterizations of the BSAIs

**Figure 6:**
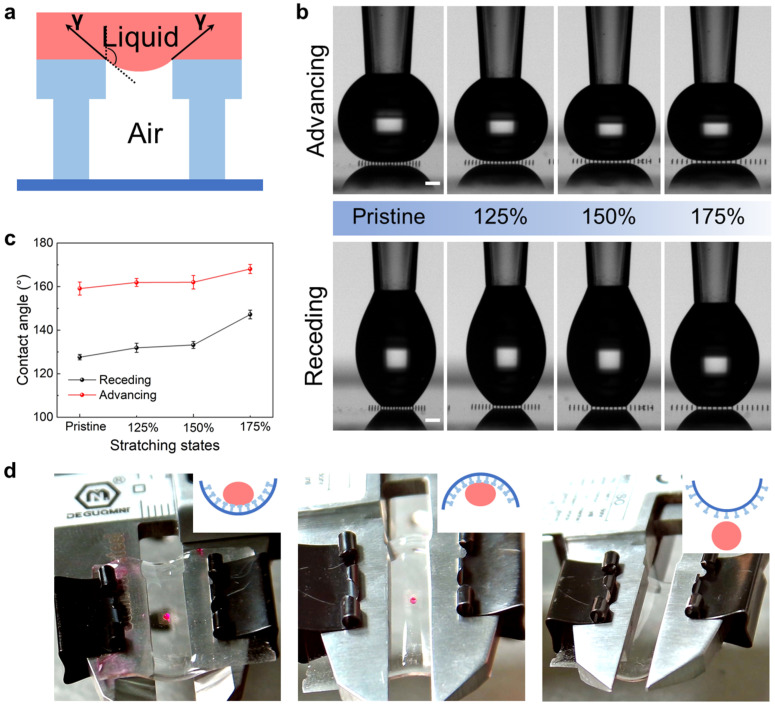
Wettability characterizations of the BSAIs. (**a**) Schematic of a water droplet suspended by the vertical component of surface tension. (**b**) Morphologies of the droplets on samples of different stretching strains. (**c**) Results of advancing and receding contact angles with related stretching strains. (**d**) Schematic and corresponding snapshots of picking up and releasing a dyed water droplet by the BSAI.

The principle of the superhydrophobic mushroom-shaped micropillars is illustrated in Figure 6a, where the vertical component of surface tension plays a predominant role. Due to the singly re-entrant structures and Young’s contact angle (*θ_Y_*) being greater than 90°, these microstructures possess the ability to support water droplets while maintaining a Cassie–Baxter state [64]. This is crucial, as once the liquid enters the Wenzel state, it becomes difficult to remove [65]. According to the Cassie–Baxter model [66,67,68], the apparent contact angle (*θ_w_*) can be expressed as follows:

$$cos\theta_{w}\approx \frac{\pi D^{2}}{4P^{2}}\left(cos\theta_{Y}+1\right)-1$$

where *D* represents the tip diameter of the micropillar, *P* denotes the patch among micropillars, and *θ_Y_* represents the intrinsic contact angle. As the *P* gradually increases, the contact area between the water droplet and the sample surface decreases, leading to a smaller liquid–solid contact fraction. The morphology of water droplets captured on the BSAI shows a noticeable decrease in the number of contacting micropillars as the sample is gradually stretched, as depicted in Figure 6b. The progressive increase in both the advancing and receding angles results in a significant reduction in contact angle hysteresis (Figure 6c), indicating that the water droplets are more easily moved from the stretched surface [69]. These findings suggest that stretching the sample creates larger gaps, leading to weaker adhesion forces on water droplets. Based on that, the BSAI demonstrates the ability to transfer liquid droplets, as illustrated in Figure 6d. Bending the BSAI downwards results in a strong adhesion force on the water droplet, preventing it from falling off. Even when the sample is flipped, the droplet remains firmly adhered to the BSAI surface. However, by reversing the deformation of the BSAI, such a droplet can be rapidly released. This switchable adhesion force to water droplets is believed to be attributed to the changes in the pitch among micropillars. Such mechanically switchable wettability and adhesion for droplets hold promising applications in the field of single-droplet manipulation, such as drug delivery [70] and droplet-based microreactors [71].

## 4. Conclusions

In conclusion, this study first fabricated mushroom-shaped PDMS micropillars on a PDMS flexible substrate by utilizing the FsLDW. Further, SEM, AFM, and optical microscopy indicated that such a method resulted in adhesion arrays with smooth tips and regular arrangement. By stretching and bending the PDMS substrate mechanically, the spacing between micropillars could be altered accurately, thereby influencing the efficient contact area. Under 175% stretching strain, the BSAI can exhibit halved adhesion force compared to the non-stretched samples. Furthermore, the variation in micropillar spacing also affected the adhesion force of liquids in the Cassie–Baxter state, enabling the rapid transfer of water droplets. This novel tactic not only overcomes the limitations of replica molding of traditional flexible adhesives, but also retains a strong connection between the micropillars and the substrate. Such advantages make them achieve a smart balance between strong adhesion and rapid switching, which hold great promise in the fields of microfluidics, micro-assembly, and beyond. Importantly, in the future, various stimuli-responsive materials can be adopted in FsLDW for the fabrication of switchable adhesive interfaces, which will largely enhance the performance of switchable adhesion and broaden their applications in interface-mediated intelligent devices or systems.

## Data Availability

The data that support the findings of this study are available from the corresponding author upon reasonable request.

## Supplementary Materials

The following supporting information can be downloaded at: https://www.mdpi.com/article/10.3390/mi14091742/s1, Figure S1: Adhesion forces of BSAIs with different heights under the same preload (20 µm).
