# Supplementary material for: Femtosecond Laser Direct Writing of Gecko-Inspired Switchable Adhesion Interfaces on a Flexible Substrate
Source: Micromachines (Basel). 2023 Sep 6;14(9):1742. doi: 10.3390/mi14091742 (PMC10534918; doi:10.3390/mi14091742)
Supplement: Supplementary file 1 [file micromachines-14-01742-s001.zip › micromachines-2576665-supplementary.pdf]

micromachines

MDPI

Supplementary Material

Femtosecond Laser Direct Writing of Gecko-Inspired Switchable Adhesion Interfaces on a Flexible Substrate

Zhiang Zhang[1], Bingze He[2], Qingqing Han[1], Ruokun He[1], Yuxuan Ding[2], Bing Han[2], and Zhuo-Chen Ma[1,2]*

[1] Department of Automation, Shanghai Jiao Tong University, Shanghai, 200240 China;
[2] Institute of Medical Robotics, School of Biomedical Engineering, Shanghai Jiao Tong University, No.800 Dongchuan Road, Shanghai 200240, China;
* Correspondence: zcma@sjtu.edu.cn (Z.-C. M.);

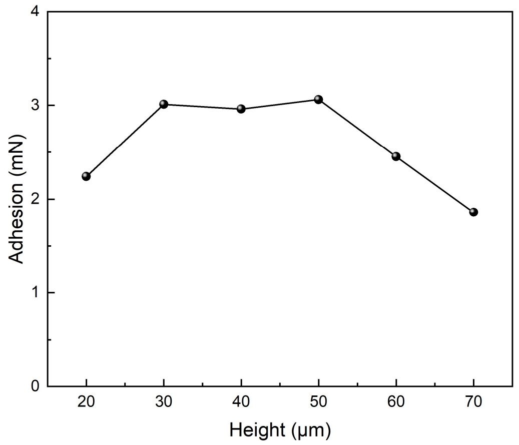

Figure S1. Adhesion forces of BSAIs with different heights under the same preload (20 μm).
